# Effectiveness and safety of the PlasmaJet® Device in advanced stage ovarian carcinoma: a systematic review

**DOI:** 10.1186/s13048-019-0545-x

**Published:** 2019-07-31

**Authors:** Gatske M. Nieuwenhuyzen-de Boer, Jacoba van der Kooy, Heleen J. van Beekhuizen

**Affiliations:** Department of Gynaecologic Oncology, Erasmus MC Cancer Institute, University Medical Center Rotterdam, P.O. Box 2040, 3000 CA Rotterdam, The Netherlands

**Keywords:** Ovarian cancer, Cytoreductive surgery, PlasmaJet®, Histology

## Abstract

About 80 % of all women affected by ovarian cancer present with advanced stage disease at the time of diagnosis. Achieving complete cytoreduction is complicated when many small tumor spots are found. Yet, complete cytoreduction is the most important determinant of survival.

Application of a thermal plasma energy device to standard surgical instruments may help achieve complete cytoreduction. The ‘PlasmaJet® Device’ (Plasma Surgical, Inc., Roswell, GA, USA) is an electrically neutral device which emits a high–energy jet of argon plasma for direct tissue effects. We performed a literature review to investigate whether the use of the ‘PlasmaJet® Device’ in surgery of advanced stage ovarian carcinoma (FIGO IIIB-IV) is effective and safe.

The primary outcome was the proportion of complete cytoreductions. The secondary outcomes were: complication rate, proportion of colostomies applied, histological findings, disease-free survival and overall survival.

Five case series or reports were found, including a total of 77 patients with FIGO stage IIIC-IV ovarian cancer in whom the PlasmaJet® device was used for primary or interval debulking. Complete cytoreduction was obtained in 79% of the patients. Apart from one pneumothorax after extensive surgery, but no harm or additional complications related to the use of the PlasmaJet® Device were reported. Data on disease-free survival or overall survival were not reported.

These findings suggest that the PlasmaJet® Device is an efficient and safe innovative surgical device for debulking surgery with encouraging results. We have proposed an RCT in which we will compare feasibility, safety and effectiveness aspects of the use of the PlasmaJet® versus conventional electrosurgery in advanced stage epithelial ovarian cancer (FIGO IIIB-IV).

## Introduction

Ovarian cancer is the fifth leading cause of cancer-related death among women and is the deadliest of gynecologic cancers worldwide [1]. Eighty per cent of all women affected by ovarian cancer present with advanced stage disease at the time of diagnosis. The standard treatment for advanced stage ovarian carcinoma is cytoreductive surgery combined with chemotherapy.

Complete cytoreductive surgery (CCS) is the most important determinant of prognosis and survival in advanced stage ovarian carcinoma [2, 3]. The success rate of the operation varies with factors such as patient selection and morbidity, tumor location and surgeon’s expertise [4]. Novel surgical and chemotherapeutic treatments introduced over the past decade have not led to significant improvement in survival.

Achieving complete cytoreduction is complicated when many small tumor spots are found on the intestines and the diaphragm. Conventional electrosurgery then often does not result in complete removal of these spots. A number of published case series suggest that application of the ‘PlasmaJet® Device’ (Plasma Surgical, Inc., Roswell, GA, USA) during cytoreductive surgery results in higher rates of complete cytoreduction and lesser need of a colostomy. The device uses neutral argon plasma to vaporize small tumor nodules with minimal collateral damage [5, 6]. This technique seems to be effective in tumor ablation; especially to remove peritoneal carcinomatosis on the abdominal peritoneum, in the diaphragmatic region, intestinal mesentery and bowel serosa.

## Methods

We performed a literature review to investigate the effectiveness and safety of the use of the PlasmaJet® device in surgery of advanced stage ovarian carcinoma (FIGO IIIB-IV) based on CT scan. We aimed to compare the outcomes of surgery with the additional use of the PlasmaJet® device with the outcomes of conventional surgery (using electrosurgery, scalpel, and scissors).

Primary outcome: percentage complete cytoreductive surgery.

Secondary outcomes: complication rate, proportion of colostomies applied, histological findings, disease-free survival and overall survival.

The following databases were searched: https://www.embase.com, https://www.controlled-trials.com, https://www.clinicaltrials.gov, and https://www.york.ac.uk/inst/crd/ at December 2018. The search strategy was as follows:

((neutral NEAR/6 argon NEAR/6 plasma) OR (jet NEAR/6 plasma NEAR/6 (coagulat* OR remov*)) OR PlasmaJet):ab,ti.

The titles and abstracts of citations retrieved from the search were screened on relevance by two authors (GN, BK) independently. Study inclusion criteria were as follows: 1) Primary epithelial ovarian, fallopian tube or peritoneal carcinoma, 2) FIGO stage IIB to IV, 3) patients treated with cytoreductive surgery, 4) residual disease categorized as complete (no macroscopic residual disease), optimal (largest diameter 0,1–1 cm) and suboptimal (largest diameter > 1 cm) and 5) complication rate reported.

## Results

The search retrieved 84 citations. After screening of titles and abstracts six articles remained. After reading of full texts by all authors, three case series and two case reports were included (Table 1). Randomized controlled trials on this topic were not found. Cordeiro et al. [7] included 51 patients with FIGO stage IIIC-IV ovarian cancer in whom the PlasmaJet® device was used for primary/interval debulking. Complete debulking was achieved in 40 (78%) patients. A pleural drain was needed in eight (16%) patients in whom the PlasmaJet® was used for diaphragmatic stripping. No other post-operative complications were found. The authors did not provide data on colostomies. Panuccio et al. [8] included 19 patients undergoing primary/interval or secondary debulking for ovarian cancer FIGO stage IIIC-IV. Complete debulking was achieved in all 19 patients. A pneumothorax occurred in one patient (5%). Bowel or urological fistulas did not occur. Renaud et al. [9] described six patients who underwent surgery with the PlasmaJet® device. Complete debulking was achieved in one patient. The authors claim that in none of these patients optimal debulking could have been reached without the use of the device. Two case studies by Seror and Butler-Manuel described the use of PlasmaJet® without any complications [10, 11].

**Table 1:** Studies using PlasmaJet for cytoreductive surgery in case of advanced stage ovarian cancer (FIGO IIIC-IV)

Author	Number	Debulking	Complete debulking	Colostomy	Complications related to PlasmaJet
CordeiroVidal G [7]	51	Primary (41%) and interval (59%)	78%	No data	Pleural drain after diafragmatic stripping (*n* = 8)
Panuccio E [8]	19	Primary and interval	100%		Pneumothorax (*n* = 1) Bloodtransfusion (*n* = 5)
Renaud MC [9]	6		20%	none	
Seror J [10]	1	Primary	Yes	no	None
Butler-Manuel S [11]	1	Interval	Yes	no	Superficial wound defect

## Discussion

The aggregated evidence from the five included studies shows that complete cytoreduction was obtained in 79% of the patients in whom the PlasmaJet® device was used during surgery. Apart from one pneumothorax after extensive surgery, no other harm or complications possibly related to the use of the PlasmaJet® device were described.

Histological examination of lateral thermal spread and the collateral tissue destruction caused by the use of the PlasmaJet® device has been performed for different power settings and exposure times. The lateral thermal spread increased with increased power, while the depth of eschar penetration remained relatively the same [4]. In a study in pig, the use of the PlasmaJet® device was compared to laparoscopic bipolar coagulation and surgical resection of the peritoneum. Histological analysis 14 days after surgery showed that all areas were equally destroyed; adhesions were only seen in bipolar coagulation [4]. Sonoda et al., investigating the thermal damage of PlasmaJet® histologically, similarly concluded that minimal lateral damage and depth of vaporization had occurred [6].

The use of the PlasmaJet® device in the removal of rectal endometriosis showed promising results with no major complications preventing colorectal resection. A randomized controlled trial in sixty women undergoing corrective abdominoplasty performed with either conventional monopolar electrosurgery or the use of the PlasmaJet® showed significantly fewer postoperative complications (mainly wound infections), one day earlier discharge, and better cosmetic outcomes with the use of the PlasmaJet® device [12].

## Conclusion

To our knowledge, this is the first systematic review on the use of the PlasmaJet® Device in surgery of advanced ovarian carcinoma. The available data suggest that the device comes with several features that are well suited for debulking surgery. Application of the device is efficient in precise tissue dissection with minimal collateral damage, especially when many small tumor spots are found on the intestines and the diaphragm.

We have proposed an RCT named PlaComOv-study in which we will compare feasibility, safety and effectiveness aspects of the use of the PlasmaJet® with those of conventional electrosurgery in advanced stage epithelial ovarian cancer (FIGO IIIB-IV) [13]. We hypothesize that the probability of achieving complete cytoreduction is significantly higher in the group of patients randomized to surgery with the use of the PlasmaJet® device. Secondary outcome include 30-days morbidity, ability to avoid bowel surgery and stoma formation, quality of life and cost-effectiveness.

## Data Availability

Not applicable.

## Abbreviations

CCS: Complete cytoreductive surgery
RCT: Randomized controlled trial

## References

[1] SEER Cancer Statistics, National Cancer Institute, https://seer.cancer.gov/statfacts/html/ovary.html

[2] Bristow RE, Tomacruz RS, Armstong DK, Trimble EL, Montz FJ (2002). Survival effect of maximal cytoreductive surgery for advanced ovarian carcinoma during the platinum era: a meta-analysis. J Clin Oncol.

[3] Du Bois A, Reuss A, Pujade-Lauraine E (2009). Role of surgical outcome as prognostic factor in advanced EpithelialOvarian Cancer. Cancer..

[4] Vitale SG, Marilli I, Lodato M (2013). The role of cytoreductive surgery in advanced-stage ovarian cancer: a systematic review. Updat Surg.

[5] Tanner EJ, Dun E, Sonoda Y, Olawaiye AB, Chi DS (2017). A comparison of thermal plasma energy versus argon beam coagulator induced intestinal injury following vaporizations in a porcine model. Int J Gynecol Cancer.

[6] Sonoda Y, Olvera N, Chi DS, Brown CL, Abu-Rustum NR, Levine DA (2010). Pathologic analysis of ex vivo plasma energy tumour destruction in patients with ovarian or peritoneal cancer. Int J Gynecol Cancer.

[7] Cordeiro Vidal G, Babin G, Querleu D, Guyon F (2017). Primary debulking surgery of the upper abdomen and the diaphragm, with a plasma device surgery system, for advanced ovarian cancer. Gynecol Oncol.

[8] Panuccio E, Leunen K, Van Nieuwenhuysen E, Neven P, Lambrechts S, Vergote I (2016). Use of PlasmaJet for peritoneal Carcinomatosis in ovarian Cancer. Int J Gynecol Cancer.

[9] Renaud MC, Sebastianelli A (2013). Optimal cytoreduction with neutral argon plasma energy in selected patients with ovarian and primitive peritoneal cancer. J Obstet Gynaecol Can.

[10] Seror J, Bats AS, Habchi H, Lecuru F (2016). Optimal surgical cytoreduction of the upper abdomen and the diaphragm for advanced ovarian cancer using PlasmaJet energy. Gynecol Oncol.

[11] Butler-Manuel S, Lippiatt J, Madhuri TK (2014). Interval debulking surgery following neo-adjuvant chemotherapy for stage IVB ovarian cancer using neutral argon plasma (PlasmaJet (TM)). Gynecol Oncol.

[12] Iannelli A, Schneck AS, Gugenheim J (2010). Use of the PlasmaJet system in patients undergoing abdominal lipectomy following massive weight loss: a randomized controlled trial. Obes Surg.

[13] Nieuwenhuyzen-de Boer GM, Hofhuis W, Reesink-Peters N, Ewing-Graham PC, van BHJ (2019). Evaluation of effectiveness of the PlasmaJet surgical device in the treatment of advanced stage ovarian cancer (PlaComOv-study): study protocol of a randomized controlled trial in the Netherlands. BMC Cancer.

